# Variants of the *EAAT2* Glutamate Transporter Gene Promoter Are Associated with Cerebral Palsy in Preterm Infants

**DOI:** 10.1007/s12035-017-0462-1

**Published:** 2017-03-07

**Authors:** Shavanthi Rajatileka, David Odd, Matthew T. Robinson, Alexandra C. Spittle, Louis Dwomoh, Maggie Williams, David Harding, Miles Wagstaff, Marie Owen, Charlene Crosby, Jared Ching, Elek Molnár, Karen Luyt, Anikó Váradi

**Affiliations:** 1 Centre for Research in Biosciences, Department of Applied Sciences, Faculty of Health and Applied Sciences, University of the West of England, Bristol, BS16 1QY UK; 2 Neonatal Neuroscience, School of Clinical Sciences, University of Bristol, St Michael’s Hospital, Southwell Street, Bristol, BS2 8EG UK; 3 Neonatal Intensive Care Unit, Southmead Hospital, North Bristol NHS Trust, Bristol, BS10 5NB UK; 4 College of Life & Environmental Sciences, University of Exeter, Stocker Road, Exeter, EX4 4QD UK; 5 Centre for Synaptic Plasticity, School of Physiology, Pharmacology and Neuroscience, University of Bristol, Biomedical Sciences Building, University Walk, Bristol, BS8 1TD UK; 6 Bristol Genetics Laboratory, Pathology Sciences, Blood Sciences and Bristol Genetics, Southmead Hospital, Bristol, BS10 5NB UK; 7 Regional Neonatal Intensive Care Unit, St Michael’s Hospital, University Hospital NHS Trust, Bristol, BS2 8EG UK; 8 Neonatal Intensive Care Unit, Gloucestershire Royal Hospital, Gloucestershire NHS Trust, Gloucester, GL1 3NN UK

**Keywords:** Brain injury, Cerebral palsy, Excitatory amino acid transporter 2 (EAAT2), Glutamate, Glutamate transporter, Neurodevelopmental disorder, Periventricular leukomalacia, Preterm infant, Promoter activity, Pyrosequencing, Single nucleotide polymorphism

## Abstract

Preterm delivery is associated with neurodevelopmental impairment caused by environmental and genetic factors. Dysfunction of the excitatory amino acid transporter 2 (EAAT2) and the resultant impaired glutamate uptake can lead to neurological disorders. In this study, we investigated the role of single nucleotide polymorphisms (SNPs; g.-200C>A and g.-181A>C) in the *EAAT2* promoter in susceptibility to brain injury and neurodisability in very preterm infants born at or before 32-week gestation. DNA isolated from newborns’ dried blood spots were used for pyrosequencing to detect both SNPs. Association between *EAAT2* genotypes and cerebral palsy, cystic periventricular leukomalacia and a low developmental score was then assessed. The two SNPs were concordant in 89.4% of infants resulting in three common genotypes all carrying two C and two A alleles in different combinations. However, in 10.6% of cases, non-concordance was found, generating six additional rare genotypes. The A alleles at both loci appeared to be detrimental and consequently, the risk of developing cerebral palsy increased four- and sixfold for each additional detrimental allele at -200 and -181 bp, respectively. The two SNPs altered the regulation of the *EAAT2* promoter activity and glutamate homeostasis. This study highlights the significance of glutamate in the pathogenesis of preterm brain injury and subsequent development of cerebral palsy and neurodevelopmental disabilities. Furthermore, the described *EAAT2* SNPs may be an early biomarker of vulnerability to neurodisability and may aid the development of targeted treatment strategies.

**Electronic supplementary material:**

The online version of this article (doi:10.1007/s12035-017-0462-1) contains supplementary material, which is available to authorized users.

## Introduction

Progress in perinatal care over the last three decades has led to greater survival rates in infants born prematurely [1, 2]. The incidence of premature birth in developed countries varies from 7.6–12% of all births [3]. While 90% of very preterm infants (below 32-week gestation) now survive beyond the postpartum period, ~35% have neurodisabilities [4]. These disabilities include cerebral palsy, cognitive- and behavioural problems [5]. The estimated cost of preterm birth throughout childhood in England and Wales with a birth rate of 700,000/year is around £3 billion per annum [6]. Susceptibility of a preterm infant to neurodisability is difficult to predict, shows considerable variation between individuals [7] and is likely to be modulated by genetic factors [8]. Better diagnostic approaches for the early identification of infants with higher risk of neurodisability are important to facilitate the development and application of appropriate treatment strategies.

Much of the neurodisability seen in very preterm infants is caused by white matter injury, known as periventricular leukomalacia (PVL) and the subsequent disruption of normal neural connectivity [9]. While the pathogenesis of PVL remains to be established, in vitro and in vivo animal studies have identified important roles for oxidative stress, cytokine-mediated injury and glutamate-induced excitotoxicity [10, 11]. Following hypoxia-ischaemia, the excitatory neurotransmitter glutamate is released into the extracellular space, causing over-activation of ionotropic glutamate receptors present in pre-myelinating oligodendrocytes [12], which induces their excitotoxic cell death and subsequent white matter lesions [10].

In the brain, neuronal and glial excitatory amino acid transporters (EAATs) play a key role in maintaining extracellular glutamate below neurotoxic levels. The activity of the predominantly astroglial high-affinity glutamate transporter EAAT2 (also known as solute carrier family 1 member 2-*SLC1A2* or the rodent ortholog glutamate transporter 1-*GLT-1*) is responsible for 90% of total glutamate uptake [13, 14]. Furthermore, EAAT2 has been implicated in the pathology of cerebral ischemia [15]. While ischaemic brain injury was exacerbated in transgenic mice lacking the EAAT2 protein in the brain [16], upregulation of EAAT2 provides neuroprotection [15]. EAAT2 is widely expressed in the white matter of the developing human brain [17] and upregulated in reactive astrocytes in post-mortem brain tissue of preterm infants with PVL, which may indicate a response to either hypoxic-ischemic injury or inflammation [18]. Collectively, these findings suggest that dysregulated EAAT2 activity may contribute to white matter damage.

A functional single nucleotide polymorphism (SNP) in the promoter region of the *EAAT2* gene has been associated with higher serum glutamate levels in adults and consequently a worse neurological outcome after stroke [19] and also with relapsing multiple sclerosis [20]. These studies raised the intriguing possibility that similar genetic differences may enhance predisposition to neurodevelopmental impairment after preterm birth. The aim of this study was to establish the role of two closely linked functional SNPs in the *EAAT2* gene promoter [19, 21] in susceptibility to brain injury and neurodisability in very preterm infants.

## Materials and Methods

### Patient Selection

The risk of CP in infants born <33 weeks of gestation is 30 times higher than among those born at term [22]. Therefore, our study included infants born at this vulnerable period. Newborns’ dried blood spots and clinical data were obtained from all infants born ≤32 weeks of gestation and survived to discharge in the South West of England recruited to the Avon Premature Infant Project (APIP; 1990–1993, *n* = 329 [23]) or received care within the neonatal unit of Gloucestershire Royal Hospital (2002–2008; *n* = 127); Southmead Hospital, North Bristol NHS Trust (2005–2010; *n* = 169) or St Michael’s Hospital, University Hospitals Bristol NHS Trust (2002–2008; *n* = 196). Infants with major congenital anomalies of the central nervous system and genetic syndromes that may cause neurodevelopmental impairment or cerebral palsy were excluded. The archived blood spots were fully anonymised according to the Human Tissue Act and Medical Research Council (UK) Guidance and used for research without individual informed consent as permitted by the UK newborn screening programme Code of Practice for the retention and Storage of Residual Spots (April 2005, ISBN 0955013801). From the total (*n* = 821 infants), 208 blood spots were not traceable, 1 was excluded with a chromosomal abnormality, ten DNA samples failed all pyrosequencing assays and 61 infants had no outcome data, leaving a total of 541 infants for the analyses (Table 1).

**Table 1:** Birth-related information and neurodevelopmental outcomes (*n* = 541). Values are numbers with % or means ± standard deviation, as appropriate. All measures were analysed independently so denominator may vary

Measure	Avon Premature Infant Project (APIP; *n* = 228)	Gloucestershire Royal Hospital (*n* = 90)	Southmead Hospital (*n* = 81)	St Michael’s Hospital (*n* = 142)
Gestational age (week)	29.9 (±2.0)	27.8 (±2.2)	26.8 (±1.8)	27.4 (±1.7)
Birth weight (g)	1435 (±384)	1130 (±347)	916 (±278)	992 (±404)
Male	131 (57.5%)	44 (49.4%)	42 (52.5%)	69 (50.4%)
Multiple birth	48 (21.1%)	27 (30.0%)	29 (36.3%)	35 (25.6%)
White ethnicity	209 (92.1%)	80 (90.9%)	–	44 (81.5%)
Apgar score				
1 min	6.3 (±2.2)	6.2 (±2.1)	5.7 (±2.1)	6.3 (±2.1)
5 min	8.5 (±1.6)	8.4 (±1.5)	7.6 (±2.1)	8.4 (±1.4)
Cerebral palsy	19 (8.3%)	12 (14.0%)	–	10 (8.4%)
Cystic PVL	18 (8.1%)	6 (6.9%)	7 (8.6%)	6 (4.4%)
Low developmental score	16 (8.0%)	9 (10.2%)	–	17 (16.4%)

### Sample Collection and DNA Isolation

Blood was collected from heel prick blood sampling on blood spot screening cards prepared routinely within 5–8 days of birth as part of the UK Newborn Screening Programme [http://newbornbloodspot.screening.nhs.uk]. DNA was isolated as described previously [24].

### Generation of Biotinylated PCR Products for Pyrosequencing

Two sequence-specific primers (EAAT2PyroF-BIO and EAAT2PyroR; Table 2) were designed to amplify a 166 bp region of the *EAAT2* promoter which included the two SNPs rs111885243:C>A or g.-200C>A (at positions -200 bp) and rs4354668:a>c or g.-181A>C (at position -181 bp) using the software provided by Qiagen Pyrosequencing. The 5′ end of the forward primer was modified with biotin. PCR reactions contained 4–6 ng of genomic DNA, 1× PCR buffer (100 mM Tris-HCl, 500 mM KCl pH 8.3), 1.5 mM MgCl_2_, 200 μM of each dNTP, 100 pmol of each oligonucleotide and 1 unit of high-fidelity Taq polymerase (FastStart High Fidelity Taq Polymerase, Roche Diagnostics Limited, West Sussex, UK) per reaction. Amplification was performed as follows: 95 °C for 5 min, 50 cycles of 94 °C for 30 s, 60 °C for 30 s, 72 °C for 30 s and final extension 72 °C for 10 min. Two additional SNPs, rs116392274 in *EAAT2* and rs1835740 [21], which are involved in glutamate homeostasis, were also analysed in the cohort and data are shown as Supplementary materials.

**Table 2:** Pyrosequencing primers and reaction conditions used in the study

Oligonucleotide	Sequence 5′-3′	Product (bp)	Annealing T (°C)	Modifications
EAAT2PyroF-BIO EAAT2PyroR	GGGGCTAAACCTTGCAATC GAGTGGCGGGAGCAGAGA	166	60	5′ Biotin None
EAAT2PyroSeq	GGGTGTGTGCGCGCC	N/A		None
Target sequence for pyrosequencing		**T/G**GGGGAGGCGGTGGAGGCC**G/T**CTG		
Nucleotide dispensation order		CGTGCAGCGTGAGCGTGC		

**Fig. 1:**
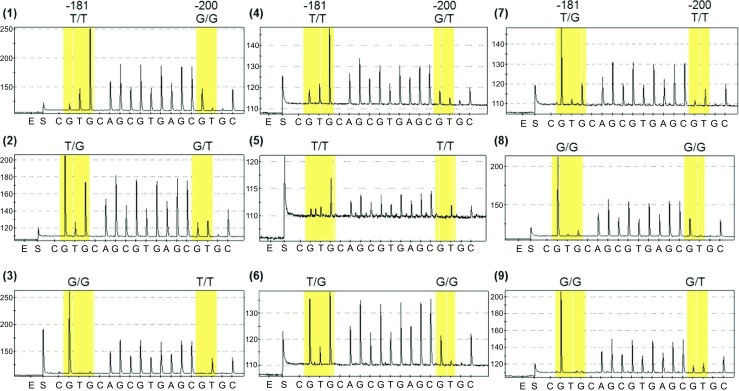
Pyrograms of the EAAT2 promoter SNPs. The position of the SNPs is highlighted in yellow boxes, the *x*-axis of each pyrogram indicates the order of reagent addition (E-enzyme, S-substrate and nucleotide A, G, T or C); the *y*-axis shows the light intensity generated. The numbering of pyrograms corresponds to the genotype numbers in Table 2. Due to the high GC content of the target sequence and the four C repeats before the SNP at position -181 bp, the pyrosequencing was carried out on the reverse strand. Thus, note that the sequence is in reverse orientation

Primer pair EAAT2PyroF-BIO/EAAT2PyroR were used to generate biotinylated PCR products flanking SNPs g.-200C>A and g.-181A>C. Primer EAAT2PyroSeq was used for pyrosequencing. The target sequence and the order of nucleotide dispensation for the pyrosequencing assay are listed. In the dispensation order the nucleotides used as negative controls are underlined. In optimal pyrosequencing conditions, these nucleotides are not incorporated into the target DNA sequence and therefore their addition do not generate peaks on the pyrogram (Fig. 1). The nucleotide change in the target sequence for pyrosequencing is indicated in bold

*N/A* not available

### Pyrosequencing and Sanger Sequencing

All steps were carried out as previously described (Table 2) [21, 24]. Genotypes of randomly selected samples (*n* = 51) from pyrosequencing were confirmed by Sanger sequencing (using ABI 3730xl 96 capillary DNA Analyzers) at Eurofins MWG Operon (Ebesberg, Germany).

### Primary Astrocyte Cultures and Preparation of the *EAAT2* Promoter Constructs

Primary rat astrocytes were separated from mixed glial cultures of embryonic (E20) Sprague-Dawley rat brains (Harlan, UK) using the previously described selective detachment (shaking) method [25]. Following separation at day 10 in vitro, astrocytes were maintained in T75 cell culture flasks (Corning Incorporated, New York, USA) at 37 °C in a humidified 5% CO_2_: 95% air atmosphere. Cells were cultured in Dulbecco’s modified Eagle’s medium (Sigma Aldrich, MO, USA) containing 4.5 g/l glucose, 29 mM sodium bicarbonate, 50 U/ml penicillin, 50 μg/ml streptomycin (Sigma Aldrich, MO, USA) and 10% (*v*/*v*) foetal bovine serum (Life Technologies Ltd., Paisley, UK). Glial fibrillary acidic protein immuno-labelling and trypan blue staining [26, 27] were used to confirm the purity and viability of the astrocyte cultures. Previously described oligonucleotides were used to amplify a 773 bp fragment of the *EAAT2* promoter [19]. Genomic DNA of genotype 1 and genotype 3 was amplified in 25 μl reactions containing 2 μl genomic DNA, 1X High Fidelity PCR buffer (100 mM Tris-HCl, 500 mM KCl pH 8.3), 1.5 mM MgCl_2_, 200 μM of each dNTP, 100 pmol of each oligonucleotide and 1 unit of high-fidelity Taq polymerase (FastStart High Fidelity Taq Polymerase, Roche Diagnostics Limited, West Sussex, UK). Amplification was performed as follows: 1 cycle at 95 °C for 5 min, 35 cycles of 94 °C for 30 s, 65 °C for 30 s, 72 °C for 1 min and final extension at 72 °C for 10 min. Following enzyme digestion and fragment purification, the promoter fragment was inserted upstream of the firefly luciferase reporter in the pGL3-basic luciferase reporter vector.

### Transfection of Astrocytes and Luciferase Reporter Gene Assay

Cells were seeded at a density of 1 × 10^5^ per well in 1 ml of complete growth medium in a 12 well plate (Corning Incorporated, New York, USA) 24 h prior to transfection. At >80% confluency, the cells were transfected using 1 μg of *EAAT2* promoter construct (EAAT2PrWT -200 bp C/C -181 bp A/A or EAAT2PrMT -200 bp A/A -181 bp C/C) and 10 μl of TransIT®-Neural Transfection Reagent (Mirus Bio, Madison, WI 5371, USA) in Opti-MEM® I Reduced Serum Media (Life Technologies Ltd., Paisley, UK). One hundred nanograms of pRL-thymidine kinase plasmid (Promega, WI, USA) containing the *Renilla* luciferase gene was co-transfected with each construct and used as an internal control. Forty-eight-hour post-transfection, the cells were washed and harvested for the promoter activity assay. All transfections were carried out in triplicates and all experiments were repeated three times. *EAAT2* promoter activity was determined using the Dual-Luciferase Reporter (DLR) Assay System (Promega, WI, USA) following the manufacturer’s guidelines.

### Patient Outcome Measures

The primary outcome measure was the diagnosis of cerebral palsy. Cerebral palsy was diagnosed when a disorder of movement and posture causing activity limitation were present at clinical examination performed at 2 years of age [28]. The secondary outcome measures were (i) cystic PVL diagnosed on a cerebral ultrasound scan during the neonatal stay and (ii) a low developmental score using standardised developmental assessment tools at 2 years of age. Cerebral ultrasound scans were performed as part of routine clinical monitoring by the clinicians in all four groups of infants. Cystic PVL was diagnosed as standard [29] (i.e. when any cystic changes were visible in the periventricular white matter on ultrasound).

Standardised developmental assessment data was available for three of the four infant groups (Table 3). The Griffith Mental Developmental Scale [30] was used for the APIP and the Gloucestershire Royal Hospital group, while the Bayley Scales of Infant Development (BSID) score (initially version II to 2006, version III after 2006 to-date) [31, 32] for the St Michael’s Hospital group. BSID-II is divided into two subscales (i) cognitive (Mental Developmental Index; MDI) and (ii) motor (Psychomotor Developmental Index; PDI). The updated BSID-III has three subscales: cognitive, language and motor PDI. Infants falling in the lowest 10th centile for either the main score (Griffith) or any of the subscales (BSID) in each infant group were defined a priori as having a low developmental score. Birth weight, gestational age at birth and physiological condition during the first 5 min after birth (Apgar scores at 1 and 5 min) were considered a priori possible confounders.

**Table 3:** Summary neurodevelopmental scores and standardised assessment used. Results are median (interquartile range-IQR). The standardised neurodevelopmental assessment scales used were: Griffith Mental Developmental Scale [30], Bayley Scales of infant development 2nd edition (BSID-II) [31] and 3rd edition (BSID-III) [32]. (For details, see the “Materials and Methods” section)

Developmental assessment used	Median (IQR)	‘Low developmental score’—10th percentile cut-off
APIP		
Griffith Mental Developmental Scale	96 (90–105)	<82
Gloucestershire Royal Hospital		
Griffith Mental Developmental Scale	101 (90–111)	<64
St Michael’s Hospital		
BSID-II-Mental Developmental Index	94 (70–108)	<51
BSID-II-Psychomotor Developmental Index	87 (71–100)	<53
BSID-III-Cognitive Developmental Index	100 (85–110)	<76
BSID-III-Language Developmental Index	96 (85–103)	<76
BSID-III-Psychomotor Developmental Index	96 (89–107)	<84

### Statistical Analysis

Initially, the perinatal/intrapartum characteristics (gestation, birth weight, gender, multiple births, ethnicity and Apgar score) of the population were assessed, split by their genotype. Then, univariable associations were assessed, between the two *EAAT2* genotypes and the primary and secondary outcome measures (see previous section). Due to the data coming from multiple infant groups with different developmental tools, multi-level logistic regression models were derived using the Stata 10 (Stata Corp, TX, USA) “xtlogit” command, to investigate the association of the odds of each additional polymorphic allele and the outcome measures. Adjustment for possible confounders was performed by adding the perinatal/intrapartum variables described above to the logistic regression models as continuous variables. Two sensitivity analyses were performed: (i) the analysis was repeated using single-level (rather than multi-level) modelling, and (ii) the missing covariates were imputed to allow the adjusted analysis to contain the same number of individuals as the unadjusted. Genotypes or outcome data was not imputed. Imputation was performed using multiple imputation with chained equations [33]. Details of imputation technique are available on request. All analyses were conducted with Stata 10 (Stata Corp, TX, USA) or Excel (Microsoft Corp, WA, US). All data are presented as odds ratio (OR) (95% confidence interval (CI)), mean (SD) or number (percent (%)).

## Results

### Simultaneous Pyrosequencing of Two SNPs in the *EAAT2* Promoter

A functional SNP was reported previously in the *EAAT2* promoter at -181 bp (rs4354668) [19]. Our detailed investigation of the *EAAT2* promoter using Sanger sequencing revealed another SNP, 19 bp upstream of rs4354668, at position -200 bp (rs111885243) [21]. These two SNPs cannot be distinguished by single-strand conformational polymorphism used in the previous study [19]; therefore, a pyrosequencing assay was developed (Fig. 1). All traceable blood spots were analysed (*n* = 613) by pyrosequencing and 521 produced clear pyrograms. Ten percent of these samples (*n* = 51) were sequenced and the concordance with pyrosequencing was 100%. In total, 471 of the infants had clinical outcome available for the analysis of rs4354668 and rs4354668.

### Distribution of Different Alleles in the Study Population

Nine genotype combinations were identified (Table 4 and Fig. 1). In 419 samples (88.9%), the two SNPs were in linkage disequilibrium (*p* < 0.001). Linkage disequilibrium was not complete and hence the nine different genotypes (Table 4). The majority of alleles demonstrated high levels of concordance, such that if the -200 locus was homozygous (C/C or A/A), the -181 locus was also homozygous (A/A or C/C) and if -200 locus was heterozygous, the -181 locus was heterozygous as well (Table 4; genotypes 1–3). In the rarer genotypes (11.0% of the cases, Table 4; genotypes 4–9), the alleles were non-concordant between the two polymorphic loci. We investigated rs116392274 (g.-168C>T) in the *EAAT2* promoter in the cohort but apart from one infant, who was a heterozygote, all others carried C/C alleles. Allele distribution of rs1835740 is shown in Supplementary materials.

**Table 4:** Distribution of genotypes in the sample cohort**.** Genotypes were identified by pyrosequencing and confirmed by Sanger sequencing (*n* = 51)

Genotype Genotype	-200C>A	-181A>C	Number and proportion
1	C/C	A/A	95 (20.2%)
2	C/A	A/C	261 (55.4%)
3	A/A	C/C	63 (13.4%)
4	C/A	A/A	9 (1.9%)
5	A/A	A/A	2 (0.4%)
6	C/C	A/C	19 (4.0%)
7	A/A	A/C	8 (1.7%)
8	C/C	C/C	1 (0.2%)
9	C/A	C/C	13 (2.8%)
Allele frequency	C = 0.56 A = 0.44	A = 0.53 C = 0.47	*n* = 471

### *EAAT2* Promoter Activity

**Fig. 2:**
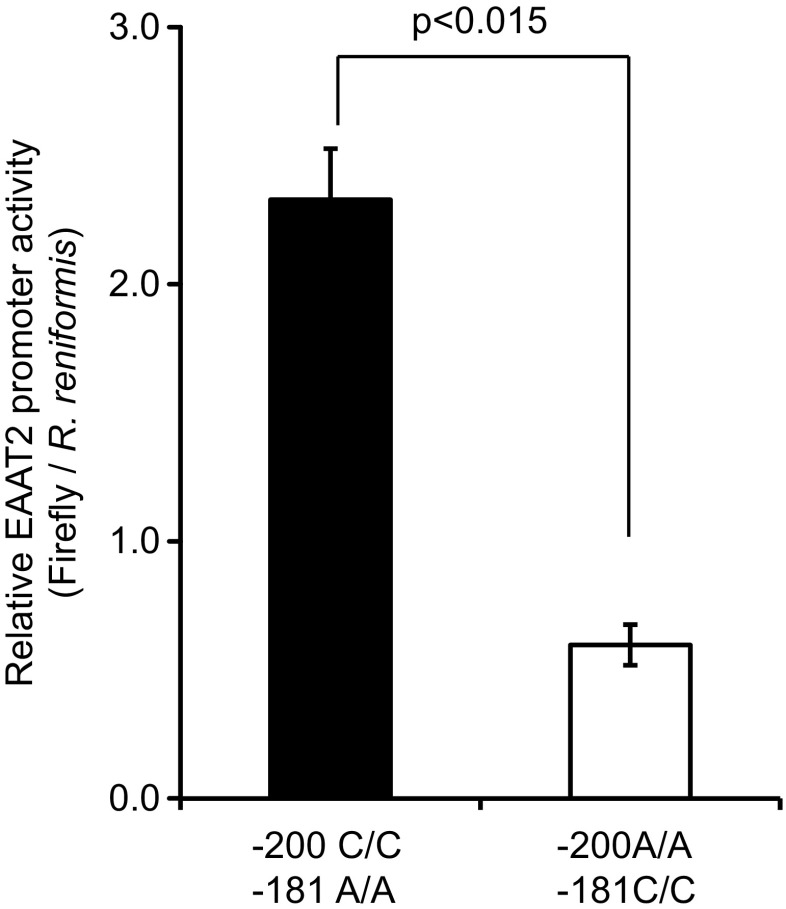
Promoter activity of *EAAT2*. Astrocytes were transiently transfected with sequences corresponding to genotype 1 (-200 C/C; -181 A/A) and 3 (-200 A/A; -181 C/C) reporter constructs. Firefly and *R. reniformis* luciferase activities were measured as detailed in the “Materials and Methods” section and the relative firefly/*Renilla* luciferase values are shown. *Bars* represent relative luciferase values from three independent experiments with standard deviation

To analyse the functional effects of the -200 C>A; -181A>C SNPs on transcriptional activity in vitro, genotype 1 (-200 C/C; -181 A/A) or genotype 3 (-200 A/A; -181 C/C) reporter constructs were transiently transfected into primary astrocytes, together with the pRL-TK vector as an internal control that constitutively expresses the *Renilla* luciferase. The genotype 1 promoter construct displayed between 4- and 4.7-fold greater activity compared with the genotype 3 construct (*p* < 0.0015; Fig. 2).

Several attempts were made to measure the promoter activity of genotypes 5 and 8 using initially the three clinical samples that carried these genotypes (Table 4). These blood samples were 15–20 years old and the isolated gDNA and the resulting PCR products were of insufficient quality [24] for successful ligation to produce the required promoter constructs. In an alternative approach, we attempted to generate these variants using site directed mutagenesis of genotypes 1 and 3. However, due to the very high GC content of the promoter amplicon (over 70%; [34]), no correct mutants were obtained.

### Characteristics of the Cohort

The intrapartum/perinatal characteristics of the eligible infants split by groups or genotypes are shown in Tables 1 and 5. Importantly, the patient outcome measures (e.g.: the rate of cerebral palsy (*p* = 0.284), cystic PVL (*p* = 0.553) and low developmental scores (*p* = 0.084)) did not differ between the four groups investigated (Table 1) and thus were combined for subsequent analysis. An association between ethnicity and genotype was observed (*p* < 0.001) when the whole cohort was investigated (Table 5). To better understand the nature of the association between ethnicity and the two SNPs, the cohort was investigated in more details. While there was no difference in the individual frequencies at the two SNPs by ethnicity (-181, *p* = 0.206 and -200, *p* = 0.854), white infants were more likely to show the concordance discussed above than non-white infants (94.6 vs. 76.1%, *p* < 0.001). Data on ethnicity was available for three of the four infant groups (Table 1) and within this population of preterm infants, there was strong evidence of deviation from the Hardy-Weinberg equilibrium (*p* < 0.001).

**Table 5:** Intrapartum/perinatal characteristics of the cohort

Perinatal measure	*n*	-200C>A			-181A>C			*p*
		CC	AC	AA	AA	AC	CC	
Gestation (week)	466	28.5 (±2.3)	28.4 (±2.4)	29.0 (±2.3)	28.6 (±2.4)	28.4 (±2.3)	28.7 (±2.5)	0.673
Birth weight (g)	466	1217 (±402)	1182 (±437)	1267 (±466)	1218 (±403)	1185 (±438)	1254 (±462)	0.690
Male	465	63 (56.8%)	147 (52.7%)	40 (54.8%)	59 (56.2%)	149 (52.7%)	42 (54.6%)	0.525
Multiple birth	466	28 (24.6%)	68 (24.4%)	22 (30.1%)	24 (22.9%)	75 (26.4%)	19 (24.7%)	0.268
White ethnicity	333	78 (91.8%)	183 (90.2%)	40 (88.9%)	77 (93.9%)	180 (90.5%)	44 (84.6%)	<0.001
Apgar score								
1 min	452	6.1 (±1.9)	6.3 (±2.2)	6.1 (±2.1)	6.1 (±2.0)	6.3 (±2.1)	6.0 (±2.2)	0.526
5 min	451	8.3 (±1.5)	8.3 (±1.7)	8.3 (±1.6)	8.3 (±1.5)	8.4 (±1.6)	8.2 (±1.7)	0.769

*n*-number of infants with data available. Values are numbers with % or means ± standard deviation, as appropriate

### Outcome Measures

In the univariable analyses (in which associations were assessed between each of the *EAAT2* SNP and the primary and secondary outcome measures independently), there was no clear evidence for an association between different alleles with cerebral palsy, cystic PVL or a low developmental score (Table 6). However, when adding both polymorphisms into the multivariable analysis, the presence of A alleles at -181 and -200 bp appeared to increase the likelihood of a low developmental score with OR of 4.56(1.53–13.60) and 3.73(1.29–10.80), respectively (Table 7; unadjusted (1)). This association persisted in the analysis adjusted for gestation, birth weight, gender and physiological condition at birth (Table 7; adjusted (2)). In contrast, there was less evidence for any association between either allele and cerebral palsy or cystic PVL. Due to the association seen with ethnicity (Table 5), this covariate was added to the model in a final adjusted analysis (Table 7; adjusted (3)). In this final model, the association with cerebral palsy strengthened with each additional A allele (locus -200 bp OR 4.34 (1.12–16.77) and locus -181 bp OR 6.64(1.76–25.07)), although there was less evidence that the polymorphism at locus -200 bp remained associated with an increased risk of a low developmental score (OR 2.84 (0.71–11.44)). Repeating the analysis using a model where the missing covariate data was imputed, the results were compatible with the main analysis. The single infant who was a heterozygote for rs116392274 had no CP or a low developmental score. Similarly, no association was observed between rs1835740 and CP or a low developmental score (Supplementary Materials).

**Table 6:** Univariable associations between genotype and outcome measures

Outcome measure	*n*	Homozygote	Heterozygote	Homozygote	*p*
-200C>A		CC	AC	AA	
Cerebral palsy^a^	385	9 (9.6%)	23 (9.7%)	3 (5.6%)	0.621
Cystic PVL	458	7 (6.3%)	21 (7.6%)	3 (4.2%)	0.566
Low developmental score^a^	349	7 (7.7%)	26 (12.4%)	3 (6.3%)	0.286
-181A>C		AA	AC	CC	
Cerebral palsy^a^	385	10 (11.4%)	23 (9.5%)	2 (3.9%)	0.263
Cystic PVL	458	7 (6.7%)	20 (7.3%)	4 (5.2%)	0.817
Low developmental score^a^	349	11 (12.9%)	22 (10.3%)	3 (5.9%)	0.424

*n*-number of infants with data available. Values are numbers with %

^a^Cerebral palsy and low developmental score data were only available from three cohorts (for details, see Table 1)

**Table 7:** Multi-level regression analysis for presence of each increasing -200 or -181 A allele and outcomes

Outcome measure	Unadjusted (1)			Adjusted (2)			Adjusted (3)		
	*n*	OR (95% CI)	*p*	*n*	OR (95% CI)	*p*	*n*	OR (95% CI)	*p*
-200C>A									
Cerebral palsy	385	1.70 (0.62–4.66)	0.299	365	1.68 (0.57–4.94)	0.346	314	4.34 (1.12–16.77)	0.033
Cystic PVL	458	0.88 (0.30–2.58)	0.812	444	0.82 (0.26–2.60)	0.740	317	0.68 (0.13–3.50)	0.641
Low developmental score	349	3.73 (1.29–10.80)	0.015	329	3.23 (1.04–10.02)	0.042	282	2.84 (0.71–11.44)	0.142
-181A>C									
Cerebral palsy	385	2.44 (0.87–6.79)	0.089	365	2.72 (0.90–8.22)	0.083	314	6.64 (1.76–25.07)	0.005
Cystic PVL	458	1.00 (0.32–3.13)	0.812	444	0.99 (0.31–3.10)	0.980	317	0.88 (0.18–4.31)	0.870
Low developmental score	349	4.56 (1.53–13.60)	0.007	329	3.93 (1.23–12.57)	0.013	282	4.15 (1.05–16.38)	0.042

(1) Multi-level for neonatal unit of care and developmental tool used. (2) Adjusted for gender, birth weight, gestation and Apgar scores at 1 and 5 min. (3) Additionally adjusted for ethnicity. *n*—number of infants with data available. Values are odds ratio (95% confidence interval)

## Discussion

### SNPs in *EAAT2* Promoter Are Associated with Neurodevelopmental Impairment After Preterm Birth

To our knowledge, this is the first study that demonstrates association between genetic variants of *EAAT2* involved in maintaining glutamate homeostasis and neurodevelopmental impairment in very preterm infants. We identified that SNP g.-200C>A in the *EAAT2* promoter is strongly linked to the previously described functional SNP g.-181A>C [19], which has not been reported in earlier studies [19, 20, 35]. The A alleles at both loci appear to increase the risk of cerebral palsy and low developmental scores (Table 7). In the common concordant inheritance pattern (Table 4, genotypes 1–3), the protective C and detrimental A alleles are usually inherited together whereas in the rare non-concordant genotypes, only detrimental alleles (Tables 4 and 8, genotypes 4/5/7) or just protective alleles (Tables 4 and 8, genotypes 6/8/9) were found at both loci. This concordance was more likely with white ethnicity. Due to the strong linkage between the two SNPs, it was appropriate to enter both into the multi-level regression analysis to assess the impact of increasing detrimental A alleles. In the multi-level regression analysis (Table 7), adjustment for gestation, birth weight, gender, multiple births and Apgar scores made no significant difference to the odds of any of the outcome measures. However, the addition of ethnicity into the regression analysis strengthened the effect seen on cerebral palsy at both loci. In addition, the odds of a low developmental score were also significantly increased with each A allele at -181 bp. To put this in context, for each additional A allele at -181 or -200, the odds of cerebral palsy increased by about four- and sixfold and the odds of a low developmental score increased fourfold. The prevalence of cerebral palsy or a low developmental score was as high as 28 and 44% for genotypes 7 and 4 with three detrimental alleles, respectively (Table 8). In contrast, no association was observed between rs116392274 or rs1835740 and CP or a low development score in the cohort indicating that these SNPs are unlikely to play important roles in the injury of the developing brain (Supplementary materials).

**Table 8:** EAAT2 genotypes and outcomes

Genotype				Low developmental score		Cerebral palsy		Low developmental score OR cerebral palsy	
A alleles	Genotype code	SNP -200 bp	SNP -181 bp	Number with outcomes	%	Number with outcomes	%	Number with at least one outcome	%
0	8	C/C	C/C	0	–	0	–	0	–
1	9	C/A	C/C	10	1 (10.0%)	10	0 (0.0%)	10	1 (10.0%)
1	6	C/C	A/C	14	0 (0.0%)	16	1 (6.3%)	16	1 (6.3%)
2	3	A/A	C/C	41	2 (4.9%)	46	2 (4.4%)	46	4 (8.7%)
2	2	C/A	A/C	192	21 (10.9%)	218	21 (9.6%)	220	32 (14.6%)
2	1	C/C	A/A	77	7 (9.1%)	78	8 (10.3%)	80	10 (12.5%)
3	7	A/A	A/C	7	1 (14.3%)	7	1 (14.3%)	7	2 (28.6%)
3	4	C/A	A/A	8	4 (50.0%)	9	2 (22.2%)	9	4 (44.4%)
4	5	A/A	A/A	0	–	1	0 (0.0%)	1	0 (0.0%)

### Regulation of *EAAT2* Promoter Activity

**Fig. 3:**
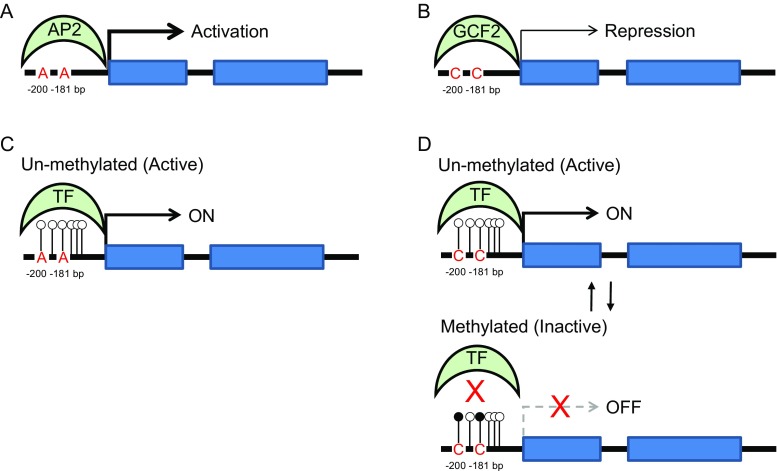
Proposed model of the SNPs impact on EAAT2 gene regulation. **a** *EAAT2* promoter contains a consensus binding site for transcription factor activating enhancer binding protein 2 (AP-2), which is an activator of transcription in the developing brain [53]. **b** Nucleotide change from A to C at -181 bp abolishes this AP-2 consensus sequence and creates a binding site for transcription factor GC-binding factor 2 (GCF2) which represses *EAAT2* gene expression [19]. **c**, **d** *EAAT2* promoter is not only controlled by the transcriptional machinery, but is also subject to modulation by epigenetic mechanism such as DNA methylation at CpG dinucleotides that inhibits gene expression [38, 39, 41, 42]. DNA methylation is reversible and subject to dynamic regulation throughout embryogenesis. Nucleotide changes from C to A might interfere with the normal DNA methylation process of *EAAT2* at both -200 and -181 bp, affecting gene expression. The ability to downregulate *EAAT2* in the developing brain seems beneficial since infants with three C alleles have better outcomes than those with only one

These two SNPs significantly affect *EAAT2* promoter activity in vitro. The promoter fragment -742/+31 [19] containing -200A/A -181C/C sequence (genotype 3) showed a 70–80% reduction in basal *EAAT2* promoter activity compared to -200C/C -181A/A (genotype 1; Fig. 2). This is a larger impact than the previously reported ~30% reduction [19]; however, in that study, the SNP at position -200 bp was not identified and it is not clear which nucleotide was present in their promoter construct. The change from A to C at -181 bp abolishes the binding site for transcription factor AP-2 (activating enhancer binding protein 2) and creates a site for GC-binding factor 2 (GCF2) which represses EAAT2 expression (Fig. 3; [19]). Reduced EAAT2 expression alters extracellular glutamate levels [13]. Despite the large difference in *EAAT2* promoter activity between genotypes 3 and 1, there was no clear association with low developmental score or cerebral palsy in any of the main three genotypes (Table 8; genotypes 1–3). Similar observations were made in patients with multiple sclerosis [20] and migraine [36] where the allele and genotype frequencies for the *EAAT2* promoter polymorphism were similar in patients and controls. However, the polymorphism at -181 bp was associated with higher plasma glutamate concentrations during relapsing multiple sclerosis [20].

Gene expression in the nervous system is not only controlled by the transcriptional machinery, but it is also subject to modulation by epigenetic mechanisms such as DNA methylation [36, 37]. Dynamic DNA methylation is observed during brain development [38, 39] and the levels of DNA methylation are increased upon ischemic injury [40]. Recent studies revealed that the basal transcriptional activity of the *EAAT2* gene is controlled by DNA methylation of cytosine residues in the region of -1010 to -1 bp of the *EAAT2* promoter [41, 42]. Hypermethylation of the *EAAT2* promoter is involved in repression of *EAAT2* activation [42]. Furthermore, a recent study revealed significant differences in the methylation of ten genes involved in neuronal and glial signalling, neurotransmission, apoptosis and cellular energetics between preterm and term infants [43]. Importantly, among these genes was *EAAT2,* which promoter was differentially methylated at multiple CpG sites. Additionally, significant variation of *EAAT2* promoter activity was observed in different brain regions and even between neighbouring cells [44]. These findings indicate that *EAAT2* promoter is dynamically regulated under physiological conditions. In genotypes 4/5/7, the C alleles at both -200 and -181 bp are replaced partially or fully by A alleles (Table 8), which might interfere with the normal methylation process and the binding of GCF2 transcription factor to the *EAAT2* promoter [19] (Fig. 3).

### Regulation of Glutamate Level by EAAT2 in the Developing Brain

One major pathology associated with cerebral palsy is PVL [10]. Oligodendrocyte cell death is particularly prominent following hypoxia-ischemia, which leads to hypomyelination [9]. Although the causes of PVL are not completely understood, cerebral ischemia is likely to play an important role [9, 10] implicating glutamate excitotoxicity, and excessive activation of ionotropic glutamate receptors [12]. The regulation of glutamate concentration in the extracellular space by EAAT2 is therefore essential for normal synaptic function [13] as well as neuronal survival by preventing excitotoxicity [16]. However, when there is a dissipation of electrochemical gradients across the plasma membrane as occurs during hypoxia-ischemia, EAAT2 operates in reverse to release glutamate, thereby promoting excitotoxicity [45]. In a rat model, glutamate was reduced in oligodendrocytes and axons following hypoxia-ischemia suggesting that these are the main sources of glutamate in developing white matter [46]. Furthermore, EAAT2 deficient mice are more vulnerable to neuronal loss in the hippocampus following a short episode of ischemia, while the wild-type mice are more vulnerable to neuronal death following prolonged ischaemia [47]. These findings suggest that in prolonged ischaemia, EAAT2 becomes the major contributor to abnormal concentrations of extracellular glutamate. EAAT2 expression is limited primarily to oligodendrocytes early in development and is increased during the period when the premature infant is most vulnerable to PVL [17]. Furthermore, the EAAT2 protein level was found to increase substantially in some cases of PVL compared to age-related controls [18]. Similarly, a recent study showed that EAAT2 is selectively expressed in cortical layer V neurons that are damaged in premature infants with PVL [48] and hypothesised that the reversal of glutamate transport by EAAT2 together with hyperactivation of ionotropic glutamate receptors contribute to excess ambient glutamate and consequently cell death specifically in these neurons [49]. Taking together, these data indicate that in the developing white matter, it is advantageous to have the ability to dynamically downregulate EAAT2 expression during ischaemia. Our genetic data supports this hypothesis; C alleles at -200 and/or -181 bp allow for dynamic alteration of EAAT2 expression via methylation and by the binding of GCF2 transcription factor (Fig. 3). In contrast, in infants who carry mainly A alleles, regulation of EAAT2 via these mechanisms is impaired, which increases ischaemic vulnerability and subsequent impaired neurodevelopment and cerebral palsy.

### Study Design Benefits and Limitations

This study included all infants of 32-week gestation or less, including multiples who survived the first 5–8 days of life. Consequently, preterm infants with severe brain injury due to hypoxia-ischaemia or intraventricular haemorrhage, who often die in the first few days of life, were not included which may explain the deviation from the Hardy-Weinberg equilibrium. Participants originated from four different infant groups/neonatal centres in the South West of England and included all ethnic groups and therefore the findings are applicable to the whole UK population of preterm infants. However, due to the retrospective design of the study, not all bloodspots could be traced from the complete population. The use of different neurodevelopmental assessment tools for the different groups precluded the use of raw cognitive or motor scores as continuous variables. The pragmatic solution was to classify those in the lowest 10th percentile of each group for each subscale/score as having a low developmental score. The lowest 10th percentile for each score translated as two standard deviations below the normal population mean, which is widely accepted as the cut-off for moderate/severe developmental impairment when using a single developmental assessment tool in clinical studies [50]. Cystic PVL was diagnosed on routine clinically directed cerebral ultrasound and white matter injury was reported and coded if it was severe and cystic in nature. We included an extra group with cystic PVL data (Table 1; Southmead Hospital) but despite increasing the power of the analysis, there was no evidence for an association between the SNPs tested and the measurable ultrasound changes. The overall proportion of cystic PVL in this work was 6.7%, which is not statistically different from the population rate in the UK Vermont Oxford dataset at the time (4.8%; *p* = 0.117). These data suggest that milder (non-cystic) white matter injury may not have been detected on clinical cerebral ultrasound in these groups and consequently an association with EAAT2 genotype and white matter damage was not found. Magnetic resonance imaging, which is more sensitive in detecting milder grades of white matter injury [51], is not used routinely in the UK to screen the preterm brain. These neuroimaging approaches performed in the first weeks of life are imprecise surrogate markers of neurological function. Therefore, structured functional neurological assessment at 2 years for cerebral palsy and neurodevelopmental impairment (used in this study) is considered to be the gold standard measure of neurological outcome in preterm infants [52].

## Conclusions

In this study, we have found that g.-200C>A and 181A>C SNPs are associated with both clinical neurodevelopmental outcomes and measurable in vitro effects on glutamate homeostasis. These findings indicate that glutamate is likely to be involved in the pathogenesis of brain injury and subsequent development of cerebral palsy and neurodevelopmental impairments in the human infant. It is plausible that g.-200C>A SNP may also have a major effect on the development of neurological diseases in the adult population as this SNP is so closely linked to the g.-181A>C SNP, which was reported to affect neurological function after adult stroke [19], multiple sclerosis [20] and in schizophrenia [35]. The described EAAT2 SNPs may have utility as a viable early biomarker of cerebral palsy and long-term neurodisability in high-risk preterm infants. These results warrant a prospective study with complete recruitment (including non-survivors) to confirm the utility as early biomarker of neurological outcome. Our results also validate the notion that glutamate plays a pivotal role in preterm brain injury and opens the debate around exploration of glutamate uptake manipulation as potential pharmacological intervention for the prevention of preterm brain injury in infants with this genetic vulnerability. Better understanding of the dynamic transcriptional regulation of EAAT2 during the perinatal period may be key to the future development of effective clinical interventions.

## Electronic Supplementary Material

Esm 1 (DOCX 64 kb)
