# Supplementary material for: Variants of the EAAT2 Glutamate Transporter Gene Promoter Are Associated with Cerebral Palsy in Preterm Infants
Source: Mol Neurobiol. 2017 Mar 7;55(3):2013–24. doi: 10.1007/s12035-017-0462-1 (PMC5840247; doi:10.1007/s12035-017-0462-1)
Supplement: Supplementary file 1 — (DOCX 64 kb) [file 12035_2017_462_MOESM1_ESM.docx]

**Supplementary material**

SNP rs1835740 is located between astrocyte elevated gene 1 (*AEG1*, also known as *MDTH*) and plasma glutamate carboxypeptidase (PGCP) both involved in glutamate homeostasis [Anttila V, Stefansson H, Kallela M, Todt U, Terwindt GM, Calafato MS, et al. (2010) Genome-wide association study of migraine implicates a common susceptibility variant on 8q22.1. Nat Genet 42:869–873]. *AEG1* downregulates the major glutamate transporter, the excitatory amino-acid transporter-2 (*EAAT2*), in the central nervous system (CNS) and *PGCP* is involved in glutamate synthesis. rs1835740 was demonstrated to be a *cis*-acting regulator of *AEG-1*, and the risk A allele was associated with higher expression levels of *AEG1* and the development of migraine and cluster headaches, suggesting a link between the rs1835740 variant and impaired regulation of glutamate levels in the CNS [Anttila V, Stefansson H, Kallela M, Todt U, Terwindt GM, Calafato MS, et al. (2010) Genome-wide association study of migraine implicates a common susceptibility variant on 8q22.1. Nat Genet 42:869–873]. Our recent work has demonstrated that in infants born at term variants of rs1835740 are associated with the need for resuscitation and the length of time the infant took to achieve a normal Apgar score [Odd D, Váradi A, Rajatileka S, Molnár E, Luyt K (2016) Association between neonatal resuscitation and a single nucleotide polymorphism rs1835740. *Acta Paediatrica* 105:e307-e312]. In infants carrying the A risk allele, the expression of *AEG-1* is expected to be elevated, which would in turn downregulate *EAAT2* resulting in elevated glutamate levels.

Due to the importance of rs1835740 in the regulation of *EAAT2*, we investigated this SNP in our preterm cohort using pyrosequencing as described previously [21] (Supplementary Table 1 and Supplementary Fig. 1). The C allele frequency in rs1835740 was 0.77 in our cohort and 0.778 in the 1,000 Genomes European Reference (p=0.73; European cohort; NCBI SNP Database; 1,000 Genome Project, Human Build 132/149; Supplementary Tables 2 and 3). There was no evidence of deviation from the Hardy-Weinberg equilibrium (p=0.159). rs1835740 did not show significant association with CP or with low developmental scores (Supplementary Tables 4-6) and therefore, it is likely that it plays no role in the injury of the developing brain.

**Supplementary Table 1. Pyrosequencing primers and reaction conditions for rs1835740**

| **Oligonucleotide** | **Sequence 5’-3’** | **Product (bp)** | **Annealing**  **T (°C)** | **Modifications** |
| --- | --- | --- | --- | --- |
| **rs1835740PyroF**  **rs1835740PyroR-BIO** | CTCATTCGTTTTCTGCCTGTTG  TCTTGCATATTTGAGCAGACTTTG | 300 | 60 | None |
|  |  |  |  | 5’ Biotin |
| **rs1835740PyroSeq** | CACAACTTGATTCCAATCT | N/A | | None |
| **Target sequence for pyrosequencing** | | G**C/T**GTATGTAGATT | | |
| **Nucleotide dispensation order** | | AGCTCGTAT | | |

Primer pair rs1835740PyroF/rs1835740PyroR-BIO were used to generate biotinylated PCR products. Primer rs1835740PyroSeq was used for pyrosequencing. The target sequence and the order of nucleotide dispensation for the pyrosequencing assay are listed. In the dispensation order the nucleotides used as negative controls are underlined. In optimal pyrosequencing conditions these nucleotides are not incorporated into the target DNA sequence and therefore their addition do not generate peaks on the pyrogram. The nucleotide change in the target sequence for pyrosequencing is indicated in bold.


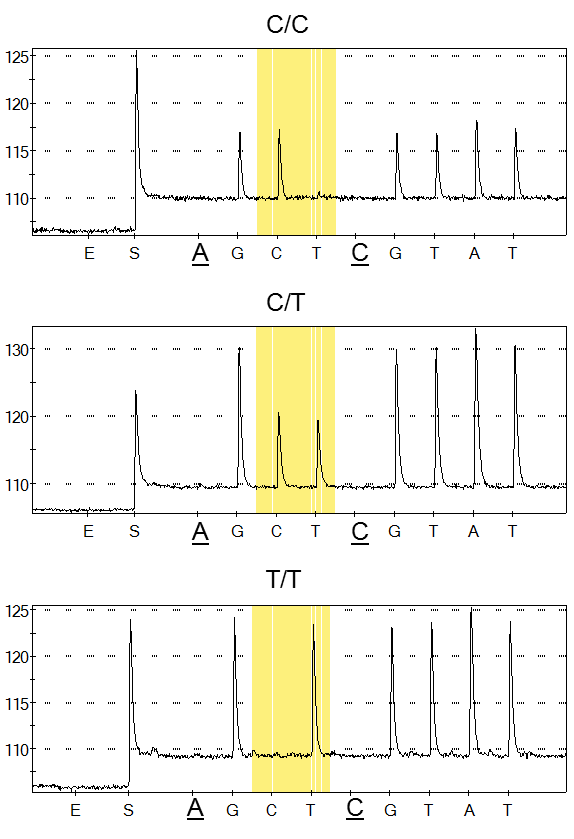


**Supplementary Fig. 1. Pyrograms of rs1835740.** The position of the SNP is highlighted in yellow boxes, the x-axis of each pyrogram indicates the order of reagent addition (E - enzyme, S - substrate and nucleotide A, G, T or C); the y-axis shows the light intensity generated. Pyrosequencing was carried out on the reverse strand. Thus, note that the sequence is in reverse orientation.

**Supplementary Table 2. Distribution of the rs1835740 alleles in the cohort.**

| **Genotype** | **Alleles** | **Number (Proportion)** |
| --- | --- | --- |
| 1 | C/C | 288 (60.0%) |
| 2 | C/T | 160 (33.3%) |
| 3 | T/T | 32 (6.7%) |
| **Allele frequency** | C=0.77  T=0.23 | n=480 |

**Supplementary Table 3. Intrapartum/perinatal characteristics of the cohort (rs1835740).** n - Number of infants with data available. Values are numbers with % or means ± standard deviation, as appropriate.

| **Perinatal Measure** | **n** | CC | CT | TT | **p** |
| --- | --- | --- | --- | --- | --- |
| Gestation (week) | 479 | 28.5 (2.3) | 28.3 (2.4) | 29.4 (2.4) | 0.062 |
| Birth weight (g) | 477 | 1196 (446) | 1148 (396) | 1290 (416) | 0.055 |
| Male | 478 | 148 (51.8%) | 93 (58.1%) | 15 (46.9%) | 0.318 |
| Multiple birth | 479 | 74 (25.8%) | 39 (24.4%) | 13 (40.6%) | 0.155 |
| White ethnicity | 332 | 178 (87.7%) | 98 (94.2%) | 25 (84.0%) | 0.137 |
| Apgar score | | | | | |
| 1 min | 468 | 6.3 (2.1) | 5.9 (2.3) | 6.8 (1.8) | 0.052 |
| 5 min | 467 | 8.3 (1.6) | 8.2 (1.8) | 8.8 (1.2) | 0.324 |

**Supplementary Table 4. Univariable associations between genotype and outcome measures (rs1835740).** n - Number of infants with data available. Values are numbers with %. ^#^Cerebral palsy and low developmental score data were only available from three cohorts (for details see Table 1)

| **Outcome measure** | **n** | **Homozygote** | **Heterozygote** | **Homozygote** | **p** |
| --- | --- | --- | --- | --- | --- |
|  |  | **CC** | **CT** | **TT** |  |
| Cerebral Palsy^#^ | 386 | 20 (8.3%) | 14 (12.1%) | 4 (13.8%) | 0.406 |
| Cystic PVL | 468 | 18 (6.5%) | 12 (7.6%) | 4 (12.9%) | 0.419 |
| Low developmental score^#^ | 347 | 24 (11.0%) | 11 (10.2%) | 3 (14.3%) | 0.858 |

**Supplementary Table 5. Multi-level regression analysis for presence of each increasing T allele (rs1835740) and outcomes.** (1) Multi-level for neonatal unit of care and developmental tool used; (2) Adjusted for gender, birth weight, gestation and Apgar scores at 1 and 5 minutes; (3) Additionally adjusted for ethnicity. n - Number of infants with data available. Values are Odds ratio (95% confidence interval).

| **Outcome Measure** | **Unadjusted (1)** | | | **Adjusted (2)** | | | **Adjusted (3)** | | |
| --- | --- | --- | --- | --- | --- | --- | --- | --- | --- |
|  | **N** | **OR (95% CI)** | **p** | **n** | **OR (95% CI)** | **p** | **n** | **OR (95% CI)** | **p** |
| **rs1835740 T>C** | | | | | | | | | |
| Cerebral Palsy | 386 | 1.39 (0.85-2.27) | 0.190 | 372 | 1.41 (0.84- 2.36) | 0.192 | 313 | 1.66 (0.96-2.85) | 0.069 |
| Cystic PVL | 468 | 1.36 (0.80-2.30) | 0.258 | 457 | 1.39 (0.81-2.40) | 0.232 | 314 | 1.33 (0.70-2.54) | 0.384 |
| Low developmental score | 347 | 1.05 (0.61-1.83) | 0.850 | 333 | 1.03 (0.57-1.88) | 0.916 | 279 | 1.23 (0.63-2.40) | 0.539 |

**Supplementary Table 6. Genotypes and outcomes (rs1835740).**

| **Genotype** | | | **Low Developmental Score** | | **Cerebral Palsy** | | **Low Developmental Score OR Cerebral Palsy** | |
| --- | --- | --- | --- | --- | --- | --- | --- | --- |
| **T alleles** | **Genotype**  **code** | **SNP** | **Number with outcomes** | **%** | **Number with outcomes** | **%** | **Number with at least one outcome** | **%** |
| 0 | 1 | C/C | 218 | 24 (11.0%) | 241 | 20 (8.3%) | 242 | 34 (14.1%) |
| 1 | 2 | C/T | 108 | 11 (10.2%) | 116 | 14 (12.1%) | 118 | 19 (16.1%) |
| 2 | 3 | T/T | 21 | 3 (14.3%) | 29 | 4 (9.8%) | 29 | 5 (17.2%) |
